# Discovery of a G-rich ultra stable human ncRNA G-quadruplex that binds ATP

**DOI:** 10.1016/j.ncrna.2026.03.005

**Published:** 2026-05-20

**Authors:** Margaret Bohmer, Kai Jin, Peixuan Guo

**Affiliations:** Center for RNA Nanobiotechnology and Nanomedicine, Center for RNA Biology, Division of Pharmaceutics and Pharmacology, College of Pharmacy, James Comprehensive Cancer Center, College of Medicine, The Ohio State University, Columbus, OH, 43210, USA

**Keywords:** ATP-Binding RNA, G-quadruplex, Noncoding RNA

## Abstract

Thermostable RNA motifs have been shown to be a unique material for building nanoparticles for many applications (Guo lab, Nature Nanotechnology, 2020 PMID: 21102465; 2021 PMID: 21909084). Here, we report the discovery of a G-rich ultra-stable human ncRNA G-quadruplex that binds ATP with a strong potential to become a drug-carrying nanoparticle. Motion is a key feature of living systems, which can be achieved with ATPase biomotors. Humans produce, metabolize, and consume ATP per day in an amount equivalent to their body weight. Due to the abundance of noncoding RNA (ncRNA) discovered in the human body, it is expected that many short or long noncoding RNAs will be involved in ATP binding and hydrolysis. This is based on the fact that, although all currently reported ATPases are proteins, only 1.5% of the human DNA genome codes for proteins, while the remaining 98.5% codes for ncRNAs that play crucial roles in regulating life functions. We discovered an endogenous human ncRNA G-quadruplex that binds ATP. This ATP-binding RNA was discovered via *in vitro* screening of endogenous ncRNAs extracted from human cells. This ATP-binding ncRNA, which was found to be G-quadruplex-forming, was characterized by ATP column binding and circular dichroism. The tightly folded, compact RNA nanoparticles will provide a new class of functional nanomaterials as both targets and carriers for drug conjugation and therapeutic delivery.

## 1 Introduction

A substantial discovery from sequencing the human genome is that while most of the genome is transcribed, only 1.5% of DNA codes for proteins. Much of the remaining 98.5%, once termed “junk DNA,” has later been found to code for short or long noncoding RNAs (sncRNA or lncRNA, respectively) that play crucial roles in cellular functioning. These functions include catalysis, scaffolding, and gene regulation.

The key feature of life is motion, and this motion is driven by the ATPase motor. ATP, which is the “currency of life,” is paramount in driving many life processes such as moving muscles or replicating through the use of biomotors. It triggers ATPases to adopt a conformation with a high affinity for binding to its substrate. After the ATPase cleaves the bond of the ATP gamma phosphate, the ATPase itself returns to its original conformation and releases the substrate [1]. This subsequently promotes the motion of biological machines [[1], [2], [3]]. On a daily basis, humans produce and metabolize ATP in an amount that is equal to their body weight, producing approximately 50–70 kg of ATP. While it has long been assumed that all ATPases are proteins, it is expected that many noncoding RNAs will be involved in ATP binding and hydrolysis [4] due to the abundance of ncRNA discovered in the human body. We sought to find human endogenous ncRNA that binds ATP. Here, that ncRNA was extracted from human cells and screened against an ATP column, resulting in the identification of a novel 41-nt ATP-binding RNA sequence derived from an intron. We also demonstrate that this ATP-binding sequence forms parallel G-quadruplexes (G4s).

## 2 Results

### 2.1 Isolation and screening of ATP-binding RNAs

**Fig. 1:**
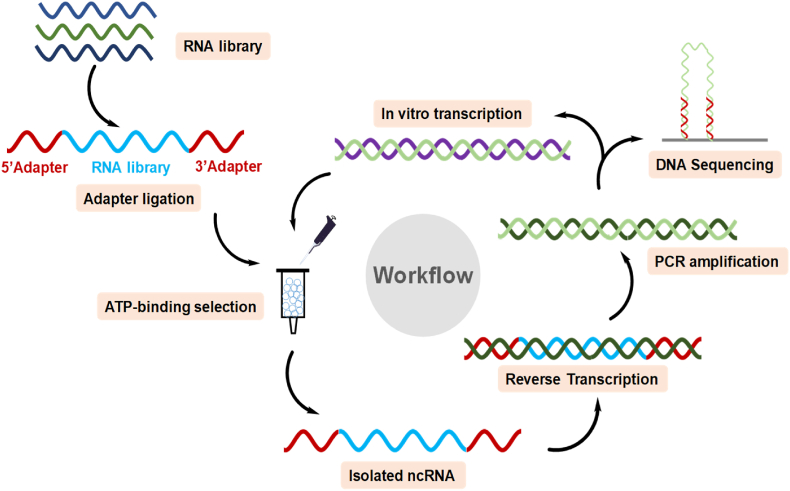
**Workflow for screening for ATP-binding RNA using intracellular RNA pool.** A total ncRNA pool was extracted from HT-29 cells, ligated with 5′- and 3′-adapters, and selected for size. A column containing ATP-resin conjugated via the C8 site was used to bind the RNA pool, which was eluted by an ATP-containing buffer. The eluted RNA was subjected to reverse transcription, PCR amplification, and transcription into a next-generation RNA pool for the next round of screening. After 4 rounds of screening, the resulting pool was sequenced.

**Fig. 2:**
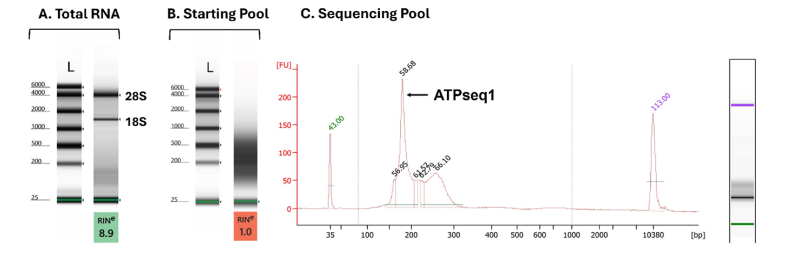
**Assessment of species in the RNA pools.** (A) Total RNA was isolated from HT-29 cells (Tapestation). (B) Starting pool for the first round of screening after removal of the rich contaminated 28S and18S ribosomal rRNA (Tapestation). L = Ladder. RIN = RNA Integrity Number. (C) The ATP-binding pool after screening round 4 prior to sequencing (Bioanalyzer).

A diagram of the *in vitro* screening process can be seen in Fig. 1. Total RNA was extracted from HT-29 cells and was confirmed to be high-quality (Fig. 2A). The RNA extracts underwent the following processing steps: rRNA removal, 5′ and 3′ adapter ligation, reverse transcription, PCR amplification, RNA transcription, and size selection (cut-off at ∼600 nts) (Fig. 2B). The 5′ and 3′ adapters served as primer-binding regions for reverse transcription and PCR amplification, as well as adapters for sequencing. The PCR amplification step included a 5′ primer that contained the T7 promoter sequence. This promoter sequence was used to transcribe the RNA pool for screening. The resulting RNA pool was applied to an ATP-agarose column linked via the C8 position of the ATP. A C8-linked ATP-agarose was chosen to avoid altering any functional groups on the ATP and because C8 modification does not affect ATP-binding of the synthetic ATP aptamer (ATP-40-1) [5]. Any RNAs that did not bind ATP were removed by passing directly through the column during the five washing steps. After washing away the non-binders, the ATP-bound RNAs were eluted with the binding buffer +5 mM ATP-Mg. Affinity elution was used in lieu of chemical or heat methods to avoid eluting RNA nonspecifically bound to the agarose matrix instead of the ATP tag. To reduce the risk of errors during reverse transcription, amplification, and transcription, excess ATP was removed from the eluted RNA with a silica spin column. The resulting RNAs were reverse transcribed and the screening cycle was repeated for a total of four rounds. The screening process was terminated after the fourth round because a sharp band was seen, indicating that one sequence was highly enriched (Fig. 2C). After the fourth round, the pool was sequenced. For more details on the screening protocol, see the Methods section.

### 2.2 Discovery and biophysical characterization of G-quadruplex structure in the ATP-binding RNA

**Fig. 3:**
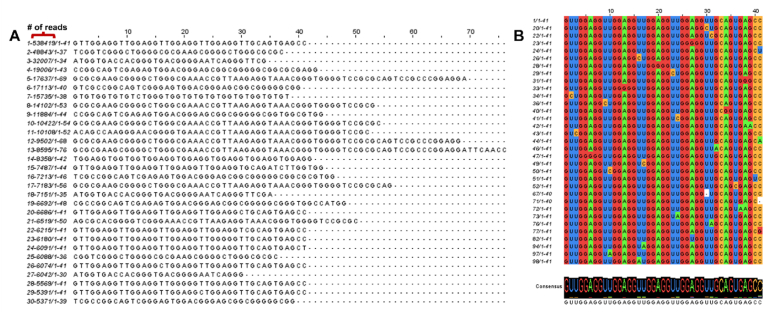
**Results from sequencing ATP-binding RNA pool.** (A) Top 30 most abundant sequences after 4 rounds of screening. The first digit of each sequence refers to the sequence number (most abundant to least abundant). (B) Multiple sequence alignment of all derivatives of ATPseq1 present within the top 100 most abundant sequences.

There was one RNA species that encompassed over 30% of the sequencing reads, which we termed “ATPseq1” (Fig. 3A, top sequence). We noticed that the simple tandem repeat region of ATPseq1 (UGGAGGU) is highly G-rich and suspected that ATPseq1 might form G-quadruplexes (G4s). These G regions were greatly enriched in the sequencing results, as none of the ATPseq1 variants in the top 100 sequencing results had mutations in the GGs (Fig. 3B). G4s are RNA or DNA oligonucleotides that contain stacks of guanines hydrogen-bonded via the Hoogsteen faces [6]. G4s can be intramolecular or intermolecular, with intramolecular G4s containing a motif of G_≥2_L_n1_G_≥2_L_n2_G_≥2_L_n3_G_≥__2_ (L refers to a linker of unspecified length) [7]. Multiple studies have revealed that G4s exist throughout the transcriptome [[8], [9], [10], [11]]. Previous NMR evidence has suggested that the (UGGAGGU)_n_ simple tandem repeat forms G4s, and inputting the ATPseq1 sequence into G4RNA, a machine-learning algorithm designed to predict the likelihood that a given sequence forms a G4, yields a G4NN value of 0.7812, which exceeds the G4 threshold of 0.5 [12].

**Fig. 4:**
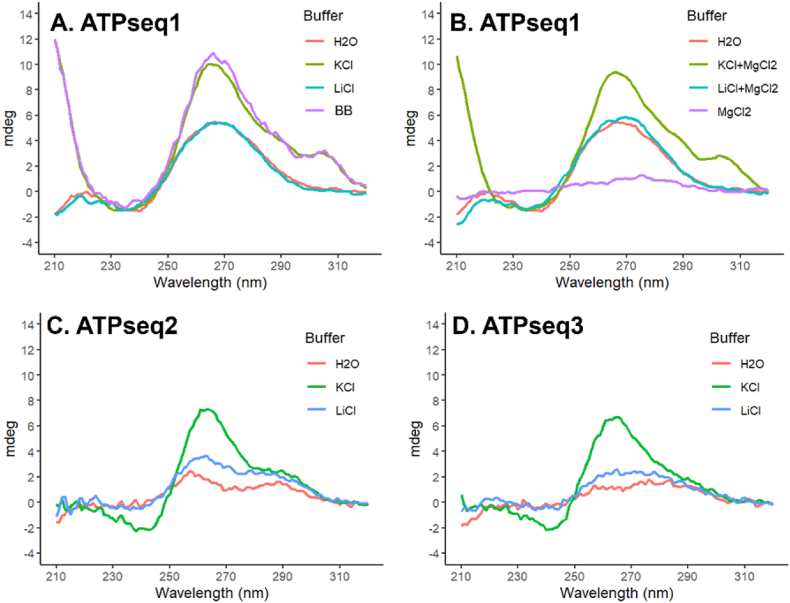
**Circular dichroism.** (A) ATPseq1 in buffer with H_2_O, 140 mM KCl, 140 mM LiCl, or 1x Binding Buffer (BB); (B) ATPseq1 in buffer with H_2_O, 5 mM MgCl_2_, 140 mM KCl + 5 mM MgCl_2_, or 140 mM LiCl + 5 mM MgCl_2_. (C) ATPseq2 and (D) ATPseq3 in buffer with H_2_O, 140 mM KCl, or 140 mM LiCl.

The capacity of ATPseq1 to form G4s was evaluated with circular dichroism (CD) spectroscopy. Parallel G4 CD spectra display a positive peak at 265 nm and a negative peak at 240 nm [13]. Since G4s form in K^+^-containing buffers but not in Li^+^-containing buffers, the CD studies were conducted with buffers containing either KCl or LiCl. As expected, the CD data show that ATPseq1 forms a parallel G4 in K^+^-containing buffer (Fig. 4A and B). ATPseq1 displayed a similar CD spectrum in a buffer identical to the one used during screening (Fig. 4A), suggesting that ATPseq1 could form G4s during the screening process. Furthermore, due to the appearance of a peak at around 305 nm, it appears that it also forms a subtype of G4 called a hexad:tetrad [[14], [15], [16], [17], [18], [19], [20], [21]]. This 305 nm peak was observed in the presence of K^+^, but not in the presence of Li^+^ or water only, indicating that K^+^ is required for the formation of the hexad:tetrad. Additionally, we also conducted CD studies in the presence of Mg^2+^, since magnesium is important for the folding of many RNAs and was present at 5 mM in the binding buffer. We observed that ATPseq1 RNA precipitated out of solution in the presence of MgCl_2_ alone, which may be an explanation for the corresponding unusual CD spectrum (Fig. 4B). This aligns with a previous publication that used high-concentration magnesium to selectively precipitate G-quadruplexes [22]. This effect was reversed when in the presence of monovalent cations (Fig. 4B), indicating that MgCl_2_ precipitates this G4-forming RNA in a monovalent cation-dependent manner. This is possibly due to M^2+^-promoted G-wire formation, which has been reported elsewhere [23,24]. When ATPseq1 was run on a native gel containing potassium, slower-moving bands were observed, which could indicate G-wire formation (Fig. S1) [25]. We compared the CD spectra of ATPseq1 and those of G-wires and found that the spectra shared an overlapping peak at 305 nm [25].

The CD spectra of ATPseq2 and ATPseq3 (second and third most abundant results from sequencing, respectively) were also obtained. When in KCl, ATPseq2 displayed a positive peak at ∼265 nm and a negative peak at ∼240 nm, indicating G4 formation (Fig. 4C). The broad peak between ∼265 and 300 nm aligns with an extended propeller loop (cite). ATPseq3 also had a positive peak at ∼265 nm and a negative peak at ∼240 nm in KCl, which was attenuated in LiCl, also indicating parallel G4 formation (Fig. 4D). These CD data suggest that both ATPseq2 and ATPseq3 are also G4-forming.

### 2.3 Identification of an ATP-binding RNA in the intron of IGF1R pre-mRNA

**Fig. 5:**
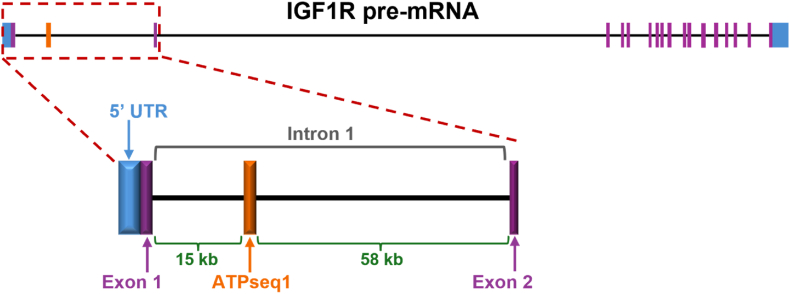
**Identification of the origin of the ATP-binding RNA.** This endogenous ATP-binding RNA, ATPseq1, was found to originate from the insulin-like growth factor 1 receptor pre-mRNA intron 1. Position: chr15:98,664,601–98,664,641; Assembly: GRCh38/hg38. The size of ATPseq1 is not to scale.

Over a third of the ∼1.5 million sequencing reads mapped to the same 41-bp region in intron 1 of the insulin-like growth factor 1 receptor (IGF1R) gene (GU(UGGAGGU)_4_UGCAGUGAGCC) on chromosome 15 (chr15:98,664,601–98,664,641; GRCh38/hg38) (Fig. 5). Notable features include a (UGGAGGU) simple tandem repeat and the final 9 nts that correspond to an AluSx Short Interspersed Nuclear Element (SINE) [26]. The (UGGAGGU)_n ≥ 3_ simple tandem repeat also occurs in intron 1 of the IQ motif and Sec7 domain ArfGEF 3 (IQSEC3) gene (chr12:91,698–91,718; GRCh38/hg38) (Fig. S2) [26]. This top sequence perfectly matches the corresponding region in the hg38 human reference genome, indicating that any ATP-binding properties are not due to mutations in the starting pool or errors during reverse transcription or PCR. Furthermore, the initial RNA pool was not fractionated prior to adapter ligation, signifying that ATPseq1 was extracted from the cells in its 41-nt form. Results 2 and 3 both originate from rRNA: 2 from 28S rRNA (residues 2904–2940) and 3 from 18S rRNA (residues 392–425).

### 2.4 Confirmation of the ATP-binding RNA

**Fig. 6:**
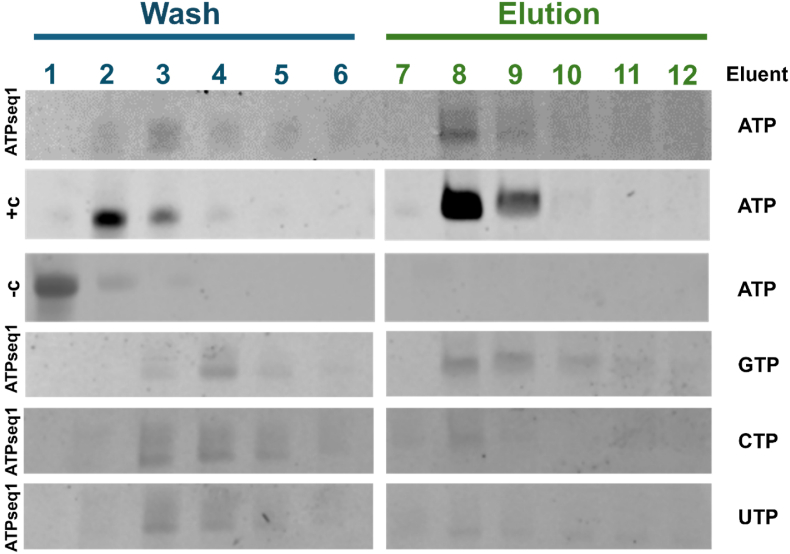
**ATP column-binding assay to verify the ATP-binding capacity of ATPseq1.** ATP-binding capacity was assessed using AT1Pseq1 synthesized via solid-state RNA synthesis. +c is ATP-40-1 (ATP-binding), and –c is ATP-40-1 G34C (non-ATP-binding). Fractions were collected before and after elution with ATP, GTP, UTP, or CTP. 8% 8M urea-PAGE gels in 1x TBE, stained with ethidium bromide.

The ATP-binding capability of the top sequence, termed ATPseq1, was tested by applying pure ATPseq1 (without the adapter sequences) to the ATP-agarose column and eluting with excess ATP. It was imperative that all subsequent studies were conducted with the 41-nt version to ensure that the observed ATP-binding is not due to the presence of the 5′ and 3′ adapter regions in the screening pool. The Sassanfar-Szostak synthetic ATP-binding aptamer (ATP-40-1) was used as a positive control. In this aptamer, G34 is required for ATP binding [4,5]. Therefore, the negative control was ATP-40-1 with a G34C mutation, which is known to not bind ATP [4]. As shown in Fig. 6, ATP successfully eluted ATPseq1 from the column, which indicates that ATPseq1 does bind ATP. It should be noted that the same amount of RNA (2 μg) was added to the column for each group (ATPseq1, ATP-40-1, ATP-40-1 G34C). The ATPseq1 trial likely has lower-intensity bands due to the documented reduced ability of ethidium bromide to stain RNA G4s [27]. The apparent K_d_ of ATPseq1 to ATP was estimated based on quantification of the gel (Fig. 6). The K_d_ (4.6 mM) was found to be extremely similar to the concentration of ATP in human cells (4.41 mM) [28]. This indicates that the ATPseq1 would be sensitive to fluctuations in intracellular ATP, which is a common feature of endogenous aptamers [29,30]. Furthermore, it is similar to the K_d_ found for the phi29 ATP-binding pRNA [4].

ATPseq1 also eluted with GTP, likely because both are purines. Minimal elution was seen with CTP and UTP elution. The ATP-binding abilities of ATPseq2 (from 28S rRNA) and ATPseq3 (from 18S rRNA) were also tested on the ATP-agarose column, but no RNA was observable in the eluant (Fig. S3). Since ATPseq2 and ATPseq3 also form G4s (Fig. 4C and D), this indicates that the ATP-binding of ATPseq1 is not due to the presence of the G4 alone.

## 3 Discussion

In this study, we selected the top three most populous results from sequencing—ATPseq1, ATPseq2, and ATPseq3—for further analysis. Additional RNAs identified in the sequencing pool include other rRNA fragments, as well as a region from the 3′ UTR of immunoglobulin superfamily member 9B (IGSF9B) mRNA, all of which we predict to form G4s based on their G-rich sequences. Further studies are required to characterize these RNAs. A limitation of this study is that it only produced RNAs that bind ATP on their own while excluding RNAs that bind to or possibly even hydrolyze ATP when in complex with a protein or cofactor. For example, our lab previously discovered that the pRNA of the phi29 phage DNA-packaging motor can bind ATP on its own but cannot participate in ATP hydrolysis unless it is bound to the DNA-packaging protein complex [4]. Another limitation is that the initial screening pool only contained RNAs that were under 600 nt (including the adapters). This was done to bypass the need to fragment the RNA pool due to the size limitations of NGS technology. This excluded RNA sequences in long RNAs, such as lncRNAs or mRNAs, from the initial pool unless those sequences existed intracellularly as degradation products.

ATPseq1 does not display any sequence or structural similarities to previously known ATP-binding RNA sequences. An ATP-binding DNA aptamer has been established to form parallel G4, but not in the hexad:tetrad conformation seen with ATPseq1 [31,32]. Furthermore, the human transcriptome-derived GTP-binding RNA also forms G4, but this RNA does not bind ATP, indicating that G4 formation alone is insufficient for ATP binding [33]. It appears that ATPseq1 most closely resembles the anti-bovine prion protein RNA aptamer, which contains (GGA) repeats and adopts a similar hexad:tetrad structure [34]. Other G4-containing RNA aptamers include ones for HIV-1 capsid, HIV-1 trans-activator of transcription (Tat) protein, human IL-6 receptor, and C9orf72 RNA (linked with amyotrophic lateral sclerosis/frontotemporal dementia) [35]. G4s are also common in fluorogenic RNAs, including Spinach, Broccoli, and Mango [36].

Both ATPseq2 and ATPseq3, although neither was established to bind ATP, were serendipitously discovered to also form G4s. ATPseq2 is derived from expansion segment 27 (ES27) in the region that formerly included helix 63 [37,38]. G4s have previously been experimentally confirmed to form in ES7 of 28S rRNA; in the same paper, the authors speculated that G4s could also form in ES27/helix 63, but this work offers the first experimental evidence of this occurring. Whole 18S rRNA is known to form G4s due to its ability to bind to BioTASQ, a G4-binding ligand, but no specific G4-forming regions in the 18S rRNA sequence had been validated until now [38]. We hypothesize that ATPseq2 and ATPseq3 were co-eluted with ATPseq1 due to quadruplex stacking [39]. The starting RNA pool had been rRNA-depleted, but according to the kit's manufacturer (New England Biolabs), 1% of rRNA remains after depletion. Therefore, it is likely that the rRNA-derived G4s were still the most abundant G4s in the starting pool, leading to their co-elution with ATPseq1.

**Fig. 7:**
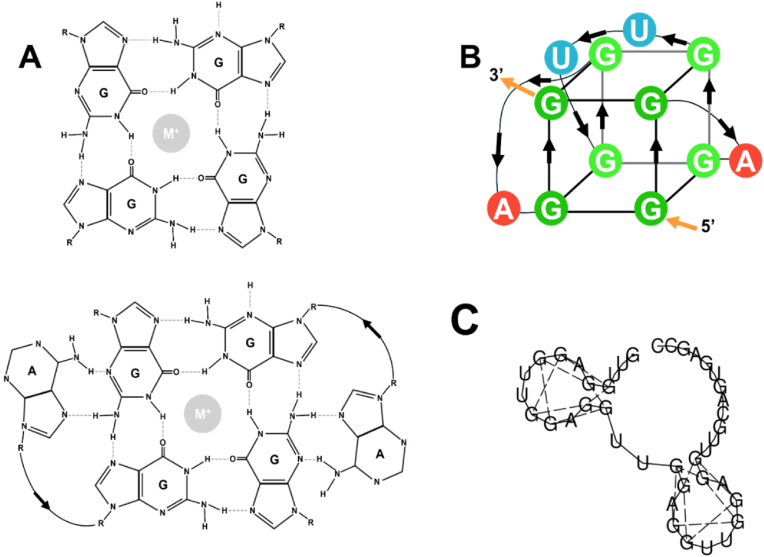
**Chemical structure of the ATP-binding RNA.** (A) The predicted G4 tetrad (Top) and hexad (Bottom). (B) 3D illustration of the hexad: tetrad G4 conformation. (C) Predicted folding of the ATPseq1 by computation (Vienna RNAfold).

ATP-binding RNA aptamers have been previously reported in synthetic pools via Systematic Evolution of Ligands by Exponential enrichment (SELEX), phi29 motor pRNA [4], and computational approaches using human genomic dsDNA in combination with SELEX (Apta-Seq) [40,41]. However, no ATP-binding RNAs had been discovered from the human transcriptome. In addition, ATP-binding RNA-quadruplex has not been reported. The causes of the difference between our findings and those reported in the literature should be further investigated. Here, we identified an ATP-binding RNA sequence derived from intron 1 of the human IGF1R pre-mRNA and discovered that this sequence forms G-quadruplexes (Fig. 7) [42]. ATPseq1 was isolated from HT-29 cells in its 41-nt form. We suspect that it was a linearized splice product that resisted degradation due to the stability conferred by the G-quadruplexes or by protection from a G4-binding protein. The region of the IGF1R intron 1 from where ATPseq1 is derived has not been linked to any phenotypes. Although it was previously stated that G4-forming sequences are generally unfolded in mammalian cells, later research found that RNA G4s can form, at least transiently, in cells and that stress promotes this folding. The 9 nts on the 3′ end of ATPseq1 are part of an AluSx SINE, and G4s have previously been found to be enriched in mobile genetic elements [43].

RNA nanoparticles are a unique and highly advantageous material for cancer treatment and RNA nanotechnology is an active field of research. Their motility and deformability enable them to penetrate the newly generated blood capillaries of tumors quickly and efficiently, leading to spontaneous tumor accumulation [[44], [45], [46], [47]]. Whole-body imaging found that most RNA nanoparticles reached the tumor environment 0.5 to 1 h after intravenous injection [44,48]. Furthermore, more than 5% of the administered dose accumulates in the tumor compared to the 0.7% accumulation reported for other types of nanoparticles [48,49]. For those RNA nanoparticles that have not entered the tumor, the negative charge of RNA nanoparticles prevents them from entering normal cells and vital organs; instead, they circulate in the blood and are quickly excreted through the glomeruli, resulting in imperceptible toxicity. The multivalent nature of RNA nanoparticles enables them to deliver multiple therapeutics for synergistic treatment of cancer [50]. Therefore, it is desirable to develop more types of RNA nanoparticles in the biomedical field in the future. The limitation of RNA nanoparticles is that they require high thermal stability (TM). Unfortunately, this type of RNA nanoparticles is currently limited [51,52]. The tightly folded ATP-binding RNA G-quadruplex reported here will provide a new class of functional nanomaterials that can serve as both targets and carriers for drug conjugation and therapeutic delivery.

## 4 Methods

### 4.1 Cell culture

HT-29 human colorectal adenocarcinoma cells (ATCC) were grown in McCoy's 5a Medium Modified (Gibco 16600082) supplemented with 10% Fetal Bovine Serum (Sigma F0926). Cells were kept in a 37 °C incubator with 5% CO_2_.

### 4.2 RNA Isolation and preparation

Total RNA was isolated from HT-29 cells using TRIzol reagent (Invitrogen 15596026). The RNA was purified using the Direct-zol RNA MicroPrep Kit (Zymo R2060) according to manufacturer instructions. rRNA was removed with the NEBNext rRNA Depletion Kit v2 (Human/Mouse/Rat) (New England Biolabs E7405S) according to manufacturer instructions. The 5′ and 3′ adapters were added using the NEXTFLEX Small RNA Sequencing Kit V4 (Revvity NOVA-5132-31). The supplementary no size selection protocol was followed. Instead of using the UDI primers from the kit, the resulting DNAs were PCR amplified with primers (obtained from IDT) for the adapter sequence. The 5′ primer included an overhang containing the T7 polymerase promoter sequence. The DNA templates were transcribed into RNA with T7 polymerase. Size selection was performed via UV shadowing and cutting of an 8% 8M urea-PAGE gel. Oligos between approximately 100 and 600 nt (after adapter addition) were cut from the gel and eluted with the crush-and-soak method in 1x Elution Buffer (0.5 M ammonium acetate, 0.1 mM EDTA, 0.1% SDS). An upper size limit of 600 nt was chosen because that is the maximum size limit of MiSeq reads. The eluted RNAs were ethanol precipitated and resuspended in nuclease-free water. The RNA was not fragmented at any point during this process.

### 4.3 In vitro *screening*

The desired amount of RNA (rounds 1 and 2: 16.8 μg, round 3: 3.6 μg, round 4: 1.8 μg) was diluted to 10 μL in water, and 10 μL 2x Binding Buffer (BB) was added. To denature and refold the RNA, it was heated in a thermocycler at 90 °C for 3 min and then slowly cooled to room temperature (1 °C every 30 s). To prepare the column, a C8-linked ATP-agarose, Adenosine 5′-triphosphate–Agarose (Sigma-Aldrich A2767), was hydrated in water and the lactose stabilizer was removed by washing under vacuum with a Buchner funnel. The hydrated resin was added to a column (Bio-Rad 732-6008) to a packed bed volume of 250 μL. The resin was equilibrated in 1x BB (10 mM Tris-HCl, pH 7.6, 140 mM KCl, 10 mM NaCl, 5 mM MgCl_2_) [40]. The 20 μL RNA sample was added to the column and immediately chased with 230 μL 1x BB. The column was washed 5 additional times with 250 μL 1x BB, with each 250 μL fraction collected separately. Following the washing steps, ATP-bound RNA was eluted with six volumes of 1x BB + 5 mM ATP-MgCl_2_. The additional MgCl_2_ was added to offset the chelating effects of ATP [53]. ATP was diluted into the elution buffer from a 100 mM stock that had been pH adjusted to pH 7 with NaOH. Each fraction was ethanol precipitated with the addition of 1 μL Glycoblue Coprecipitant (Invitrogen AM9515). After resuspension in 10 μL water, excess ATP was removed with the GeneJET RNA Cleanup and Concentration Micro Kit (Thermo Scientific K0841) according to manufacturer instructions. Half of each elution fraction (5 μL) was combined (30 μL total from 6 elution fractions) for excess ATP removal. Excess ATP removal is an essential step—our earlier attempts skipped this step and it resulted in dramatically reduced PCR product yield by the second round. The RNA was then ethanol precipitated again with Glycoblue and resuspended in 10 μL nuclease-free water, 8 μL of which were used for reverse transcription with the Superscript III First-Strand Synthesis System (Invitrogen 18080051) using a primer for the 3′ adapter sequence. For PCR, 8 μL of the reverse transcription reaction product was amplified with the same primers used for the initial DNA template replication. The DNA templates were transcribed back into RNA, treated with DNase I, gel-purified, and the cycle was repeated.

### 4.4 Sequencing

Following four screening rounds, the RNA pool was reverse transcribed and PCR amplified with primers lacking the T7 polymerase promoter. The DNA product underwent another PCR using the NEXTFLEX Small RNA Sequencing Kit V4 UDI barcoded primer mix 1. The library was sequenced with a MiSeq machine (Illumina) and a MiSeq Reagent Kit V2 (300 cycle) (Illumina). To account for the low library diversity resulting from the screening process, 5% PhiX Control (Illumina FC-110-3001) was used instead of the standard 1%. The sequencing data were analyzed on the Galaxy web server (usegalaxy.org) [54]. The following pipeline was used: 1. Filter by quality (filter out low-quality reads), 2. FASTQ to FASTA, 3. Clip (remove adapter sequences), and 4. Collapse (combine identical reads and sort by abundance). Locations of sequences in the human genome were identified via BLAT searches on the UCSC Genome Browser [26,55]. Sequences were aligned with Jalview [56].

For the cell culture assay, the total RNA samples were rRNA-depleted with the QIAseq FastSelect -rRNA HMR Kit (Qiagen 334386) and the libraries were prepared with the QIAseq Ultralow Input Library Kit (Qiagen 180497). The libraries were sequenced on an Illumina NovaSeq X with the NovaSeq X Series 10B Reagent Kit (300 Cycle) (Illumina 20085594).

### 4.5 Confirmation of ATP binding

ATPseq1 RNA was obtained from IDT. ATPseq2, ATPseq3, ATP-40-1, and ATP-40-1 G34C RNAs were synthesized in-house via phosphoramidite chemistry using an ASM-800ET DNA/RNA synthesizer (Biosset). For confirmation of ATP binding, 2 μg of RNA was added to a 250 μL ATP-agarose column, the same type that was used in the initial screen. The RNA was washed and ATP-eluted using the same protocol from the screen. For GTP, CTP, and UTP elutions, the ATP in the elution buffer was replaced with the specified NTP. Each fraction was ethanol precipitated with 1 μL Glycoblue Coprecipitant and resuspended in 10 μL water. Four μL of each fraction was run on a 16% 8M urea denaturing PAGE gel in 1x TBE. The gels were stained with ethidium bromide and visualized. For ATPseq1 elution with ATP, the gel bands were quantified with ImageJ [57] and used to calculate the K_d_ = $\frac{\left[R\right]\left[L\right]}{\left[RL\right]}$, where [R] = unbound ATPseq1, [L] = concentration of ATP on the column (3 mM), and [RL] is ATPseq1 that bound ATP on the column.

### 4.6 Circular dichroism

Samples of 10 μM RNA in the respective buffer were prepared, heated to 90 °C for 3 min, slowly cooled to room temperature, and then put into a 1 mm quartz cuvette (Hellma Analytics 110-1-40). CD measurements were taken using a Jasco J-1500 Circular Dichroism Spectrometer with the following parameters: 200 mdeg/0.1 dOD CD scale, 2 s digital integration time, 1 mm bandwidth, 1 nm data pitch, continuous scanning, and 100 nm/min scanning speed. All CD data presented were auto-corrected to a buffer-only baseline and averaged from three measurements per sample.

## CRediT authorship contribution statement

**Margaret Bohmer:** Writing – review & editing, Writing – original draft, Visualization, Methodology, Investigation, Formal analysis, Data curation. **Kai Jin:** Resources. **Peixuan Guo:** Writing – review & editing, Writing – original draft, Supervision, Project administration, Funding acquisition, Conceptualization.

## Declaration of competing interest

The authors declare the following financial interests/personal relationships which may be considered as potential competing interests: P.G. is a co-founder of ExonanoRNA, LLC and a consultant of RNA Nanobiotechs.

## Data Availability

Data supporting the findings of this study are available within the article and its supplementary materials or are available upon request.
