# Supplementary material for: Discovery of a G-rich ultra stable human ncRNA G-quadruplex that binds ATP
Source: Noncoding RNA Res. 2026 May 20;19:108–15. doi: 10.1016/j.ncrna.2026.03.005 (PMC13213811; doi:10.1016/j.ncrna.2026.03.005)
Supplement: Multimedia component 1 [file mmc1.docx]

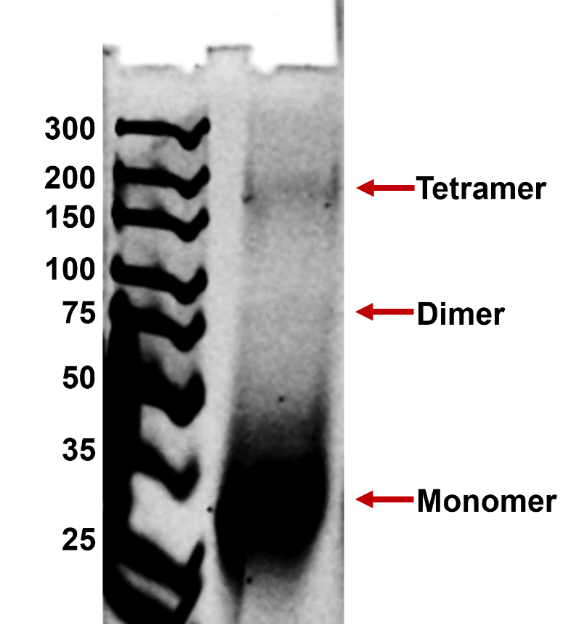


**Figure S1: Potential G-wire formation in ATPseq1.** Lane 1: DNA ladder. 2: ATPseq1 (200 ng). 16% native-PAGE gel in 1x TBM + 140 mM KCl.

**
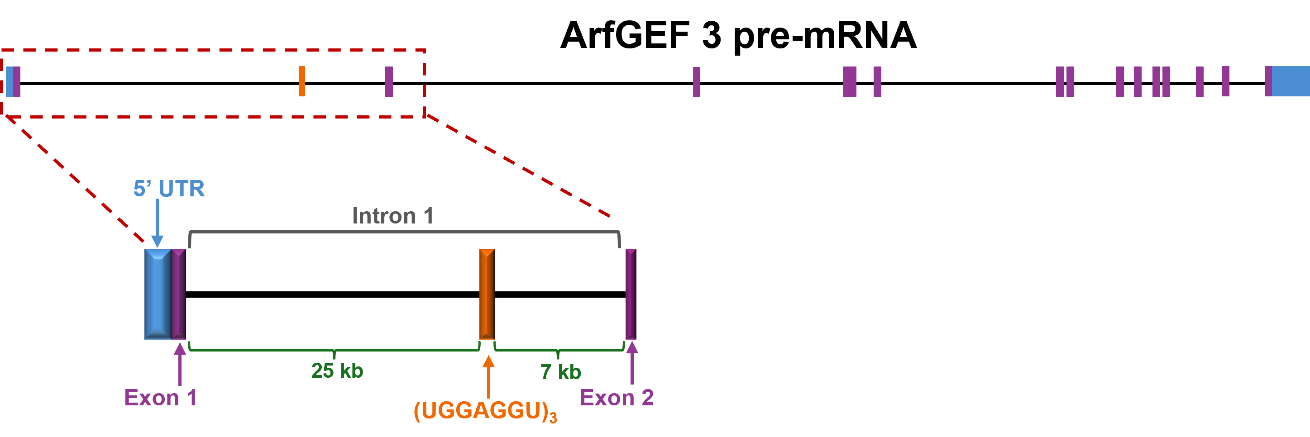
**

**Figure S2: Identification of the UGGAGGU motif in ArfGEF 3 pre-mRNA.** The UGGAGGU motif found in ATPseq1 was also identified in IQ Motif and SEC7 Domain-containing Protein 3 pre-mRNA. Position: chr12:91,698-91,718; Assembly: GRCh38/hg38. Sizes are not to scale.

**
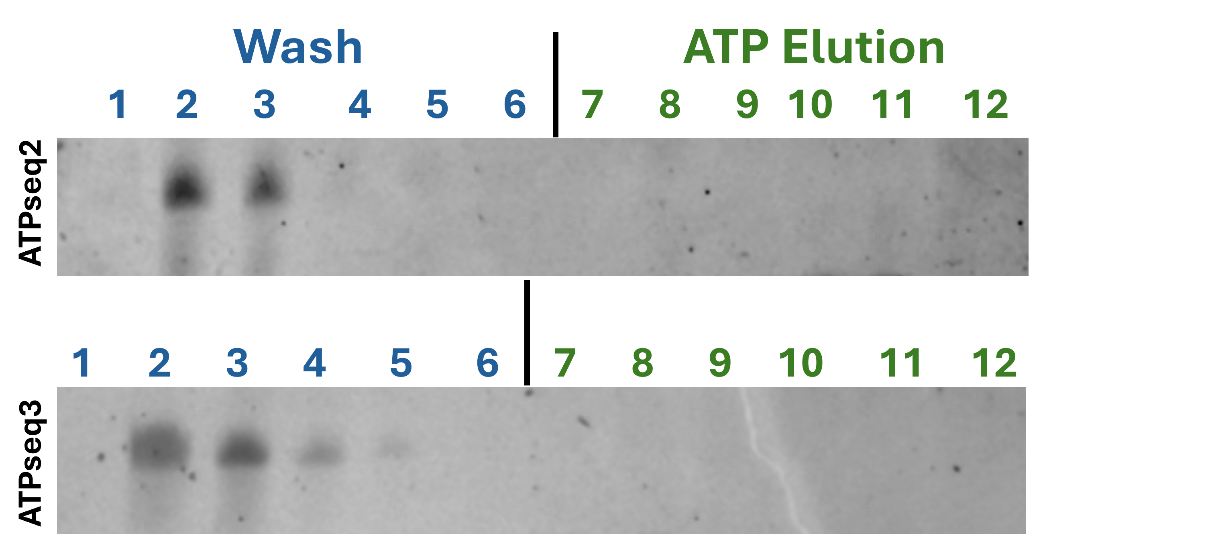
**

**Figure S3: ATP column-binding assays** of ATPseq2 (Top) and ATPseq3 (Bottom). Fractions were collected before and after elution with ATP. 8% 8M urea-PAGE gels in 1x TBE, stained with ethidium bromide.
